# Epigenetic modulations of noncoding RNA: a novel dimension of Cancer biology

**DOI:** 10.1186/s12943-020-01159-9

**Published:** 2020-03-24

**Authors:** Xiao Yang, Ming Liu, Mengmeng Li, Sen Zhang, Hong Hiju, Jing Sun, Zhihai Mao, Minhua Zheng, Bo Feng

**Affiliations:** 1 Department of General Surgery, Division of gastrointestinal and colorectal Surgery, Ruijin Hospital, Shanghai Jiao Tong University School of Medicine, Shanghai, 200205 China; 2 Department of genecology and obstetrics, Tongren Hospital, Shanghai Jiao Tong University School of Medicine, Shanghai, 200205 China; 3 Shanghai tenth People’s Hospital, Medical School of Tongji University, Shanghai, 200205 China

**Keywords:** RNA epigenetics, Noncoding RNA, Cancer progression, Cancer treatment

## Abstract

Empowered by recent advances of sequencing techniques, transcriptome-wide studies have characterized over 150 different types of post-transcriptional chemical modifications of RNA, ranging from methylations of single base to complex installing reactions catalyzed by coordinated actions of multiple modification enzymes. These modifications have been shown to regulate the function and fate of RNAs and further affecting various cellular events. However, the current understanding of their biological functions in human diseases, especially in cancers, is still limited. Once regarded as “junk” or “noise” of the transcriptome, noncoding RNA (ncRNA) has been proved to be involved in a plethora of cellular signaling pathways especially those regulating cancer initiation and progression. Accumulating evidence has demonstrated that ncRNAs manipulate multiple phenotypes of cancer cells including proliferation, metastasis and chemoresistance and may become promising biomarkers and targets for diagnosis and treatment of cancer. Importantly, recent studies have mapped plenty of modified residues in ncRNA transcripts, indicating the existence of epigenetic modulation of ncRNAs and the potential effects of RNA modulation on cancer progression. In this review, we briefly introduced the characteristics of several main epigenetic marks on ncRNAs and summarized their consecutive effects on cancer cells. We found that ncRNAs could act both as regulators and targets of epigenetic enzymes, which indicated a cross-regulating network in cancer cells and unveil a novel dimension of cancer biology. Moreover, by epitomizing the knowledge of RNA epigenetics, our work may pave the way for the design of patient-tailored therapeutics of cancers.

## Background

Over the past decade, driven by technological advances and novel mechanistic insights, we are witnessing giant strides in cancer research and treatment. However, the prognosis of advanced cancer patients is still dismal [1]. Two major reasons account for this are supposed to be the postsurgical recurrence and low response to chemotherapeutic agents. Therefore, a better understanding of the molecular mechanism of cancer progression and chemoresistance is still urgently needed.

Similar to conventional epigenetic modification of DNA and histones, diverse chemical modifications of cellular RNAs (termed as ‘RNA epigenetics’ or “epitranscriptome”) [2, 3], including N6-methyladenosine (m6A) [4], N1-methyladenosine (m1A) [5], 5-methylcytidine (m5C) [6] and 7-methylguanosine (m7G) [7], have emerged as pivotal regulators of gene expression. These epigenetic marks expand the chemical and topological properties of four basic nucleotides, thereby affecting the structures and functions of RNAs [8]. For decades, research on RNA epigenetics mainly focused on transfer RNAs (tRNAs) and ribosomal RNA (rRNA) due to their high abundance and intensive modulation [9, 10]. In the last couple of years, the development of sequencing techniques and methodology made it feasible to shift the scope of research field towards transcriptome-wide range. As the most prevalent internal modification of RNA in eukaryotic cells, m6A is now being pushed to the front of the molecular biology for the discoveries of its functional importance in various fundamental bioprocesses and components named “writers”, “erasers”, and “readers” which install, remove, and recognize the m6A residues along the target RNA transcripts [11–13]. Similar to m6A, other RNA modifications such as m1A, m5C and m7G are also highly dynamic and some are reversible, forming a new layer of the post-transcriptional regulatory landscape [3, 5]. Moreover, it is becoming evident that the alterations of the RNA modification machinery can have detrimental effects in human diseases, especially in cancers [14].

Accumulating evidence from human genome sequencing and annotations has revealed that only 2–3% of the human genome constitutes protein coding genes, while most of the genome transcribed as noncoding RNAs [15, 16]. MicroRNAs (miRNA) are small, 19- to 22-nucleotide sequences of noncoding RNAs which inhibit translation or result in the degradation of target mRNA by binding to the 3′-untranslated regions (3′-UTR) [17]. In contrast, lncRNAs are transcripts with lengths greater than 200 nucleotides and limited protein coding capacities [18, 19]. Unlike above two linear RNAs, circRNA is a type of single-stranded RNA that forms a covalently closed continuous loop [20]. As the 3′ and 5′ ends normally presented are linked, circRNAs are insensitive to ribonucleases [21]. In recent years, numerous studies have identified that ncRNAs participate in various cellular biological processes such as cell cycle, apoptosis and differentiation by acting as signals, scaffolds, molecular decoys and sponges. Malfunctions of ncRNAs have been found to be involved in the initiation and progression of most of human malignancies [22, 23].

Based on the results of previous studies, researchers presumed that epigenetic modulation of ncRNAs might represent as a novel mechanism of cancer progression. To date, this hypothesis has been validated in several kinds of cancer [24–28]. As the most abundant RNA modulation, m6A was detected in more than 7000 mRNAs and hundreds of lncRNAs in human transcriptome [8, 29]. Notably, m6A sites seem to be more enriched in ncRNAs (78 sites at most) compared with mRNA (3–5 m6A sites per mRNA) [29, 30], suggesting the potential importance of m6A in regulating ncRNA. In this review, we summarized the epigenetically modified ncRNAs and their functions and therapeutic potential in human cancer. By reviewing this, we aim to show a novel dimension of epigenetics and lay a theoretical foundation for the design of patient-tailored target therapy.

### N6-methyladenosine (m6A)

#### Major components of m6A

M6A is the most prevalent internal RNA modification generally lie within a RRACH [31] or DRACH [32] sequence motif (R = A or G; H = A, C or U) in proximity to stop codons and 3’untranslated region [4]. Functionally, m6A have been shown to participate in almost every aspect of RNA metabolism and function including RNA stability, alternative splicing, mRNA translation, secondary structure formation, and subcellular location [30, 33–35]. Being precisely controlled by several “writers”, “erasers”, and “readers”, the levels of cellular m6A are highly dynamic and reversible, suggesting the possibility that m6A may serve important functions in cell signaling networks.

The installation of m6A is catalyzed by the m6A methyltransferase complex (MTC) composed of two “writer” proteins, methyltransferase-like 3 and 14 (METTL3 and METTL14) [11, 36]. METTL3 is identified as an active S-adenosylmethionine (SAM)-dependent methyltransferase whereas METTL14 appears to be a pseudomethyltransferase mainly contributes to recognition and binding of target transcripts [37–39]. The METTL3-METTL14 complex is the principal m6A forming enzyme which targets a wide spectrum of RNA substrates [36, 39]. However, numerous m6A modifications are mapped within different sequence contexts not detected in the MTC crosslinking immunoprecipitants, indicating other m6A methyltransferases may exist in human cells [32]. Recently, METTL16 has been characterized as another m6A “writer” protein. Target spectrum analysis uncovers the interactions between METTL16 with various mRNAs, lncRNAs and U6 snRNA [40–42]. Interestingly, in contrast to METTL3-METTL14 mediated m6A methylation, the majority of METTL16 crosslinking sites were found in ACm6AGAGA motif located mostly in introns, implying distinct subsets and diverse functions of m6A modifications [40]. Considering the association between ACm6AGAGA sequence and spliceosomes, researchers presume that METTL16 may play an important role in RNA alternative splicing [41].

The formation of the MCT needs adaptor proteins to act as “scaffold”. Wilms tumor 1-associated protein (WTAP) is the first identified adaptor protein which interacts with METTL3, METTL14 and many other proteins and RNAs, suggesting WTAP may recruit other factors and target RNAs to m6A complex [38, 43, 44]. Other adaptor proteins such as RNA-binding motif protein 15 (RBM15), RBM15B and were proved to be interacted with MTC in a WTAP-dependent manner [29, 45]. Depletion of these adaptor proteins disintegrated the m6A complex and diminished the global cellular m6A levels. Once regarded as accessories of m6A complex, WTAP was proved to promote progression of hepatocellular carcinoma and ovarian cancer [46, 47]. The discovery of the fat mass- and obesity-associated protein (FTO) as the first RNA demethylase reinforces the idea that m6A is dynamic and reversible process analogous to DNA and histone modifications [13, 48]. FTO can sequentially oxidize m6A to N6-hydroxymethyladeosine (hm6A) or N6-formyladenosine (f6A), which are unstable and can be hydrolyzed to adenine. Another m6A eraser protein, alkylation repair homolog protein 5 (ALKBH5), catalyzes the direct removal of methyl group from adenine [49]. Several recent studies highlighted the vital roles of FTO and ALKBH5 in cancer progression by regulating the stability and functions of some key ncRNAs.

Like DNA and histone methylation, m6A is also an epigenetic mark which needs to be decoded by m6A “reader” proteins to generate a functional signaling. YT521-B homology (YTH) domain-containing protein family is the most predominant m6A readers and directly bind to m6A modified RNA bases. YTHDF1 is involved in regulating translation efficiency whereas YTHDF2 is proposed to affect RNA stability [50–52]. YTHDC1 is characterized as a regulator of RNA splicing and mediated the X-chromosome silencing effect of lncRNA *XIST* [29, 53]. Another m6A reader protein is eukaryotic initiation factor 3(eIF3), which is recruited by m6A residues located in the 5’UTR of mRNA and mediates cap-independent translation [54, 55]. In contrast, members of heterogeneous nuclear ribonucleoprotein (HNRNP) family, including HNRNPA2B1 and HNRNPC, seem to choose their target transcripts by screening the RNA binding motifs (RBMs) which are more accessible to them as a result of m6A modification [35, 56]. This mechanism is termed as “m6A switch”, which means m6A alters the local structure of mRNA or lncRNA to facilitate binding of HNRNPs for biological regulation [57]. Other m6A readers include insulin like growth factor 2 mRNA binding protein (IGF2BP) family were reported to regulate stability of m6A methylated RNAs [25].

Collectively, m6A is precisely controlled by various “writers”, “erasers”, and “readers” and plays a vital role in RNA metabolism, especially in processing of miRNAs and functions of lncRNAs.

#### m6A regulates processing of miRNAs

MicroRNAs are broadly conserved small RNAs implicated in a wide range of pathological processes, including cancer initiation and progression [17]. Previous studies discovered that m6A was involved in the processing of miRNAs by interacting with the miRNA processor protein DGCR8 under physiological conditions [58]. Notably, similar mechanism was observed in cancer cells. A recent study assessed the association between METTL3 and DGCR8 in a bladder cancer model. Results showed that METTL3 promoted proliferation of bladder cancer cells both in vitro and in vivo by enhancing the binding of DGCR8 to pri-miRNA-221/222 through its m6A activity, which resulted in the accelerating maturation of pri-miR-221/222 and reduction of tumor suppressor PTEN, the target of miR-221 [59]. Moreover, METTL3 also stimulated the maturation of miR-1246 and thereby downregulated tumor suppressor gene SPRED2, leading to enhanced metastatic capacity of colorectal cancer cells [60]. Further mutation assay in which adenine in the m6A motif was replaced with guanine confirmed this activity of METTL3 was m6A-dependent. In addition, METTL14 could interact with DGCR8 in a manner similar to METTL3 and positively modulated the primary miR-126 processing to suppress the metastasis of hepatocellular carcinoma [61]. Besides, METTL3 promoted the malignant transformation in bronchial epithelial cells by upregulating miR-106b and miR-18b, which were closely associated with cell proliferation and apoptosis [62]. However, the potential mechanisms by which METTL3 regulate these miRNAs were not addressed.

The two key demethylases, FTO and ALKBH5, were also involved into the regulation of cancer progression as mRNA handlers. FTO acted as an oncogene in acute myeloid leukemia (AML) through regulating target genes such as *ASB2* and *RARA* by reducing m6A levels in these mRNA transcripts [63]. ALKBH5 promoted renewal and growth of breast cancer cells by reversing m6A of *NANOG* mRNA, which in turn enhanced the stability of *NANOG* and stemness of cancer cells [64]. In terms of miRNA regulating, research of ALKBH5 seemed to go one step further. ALKBH5 was shown to regulate the processing of miR-7 in a HuR dependent manner in ovarian cancer and to affect the expression of miR-7 target gene EGFR, which gave us a hint that ALKBH5 might serve as a biomarker or target in the anti-EGFR chemotherapy [65]. FTO was proved to be negatively regulated by miR-1266 in colorectal cancer cells [66], but the effect and mechanism of FTO or miR-1266 on cancer progression was not addressed. FTO affected the expression of miR-130, miR-155 and miR-378 involved in brown adipogenesis [67], suggesting the existence of the cross-regulation network between FTO and miRNAs and the possibility that FTO might target miRNAs to regulate the phenotypes of cancer cells. However, the molecular mechanisms by which FTO interacts with miRNAs need to be investigated in detail.

**Fig. 1:**
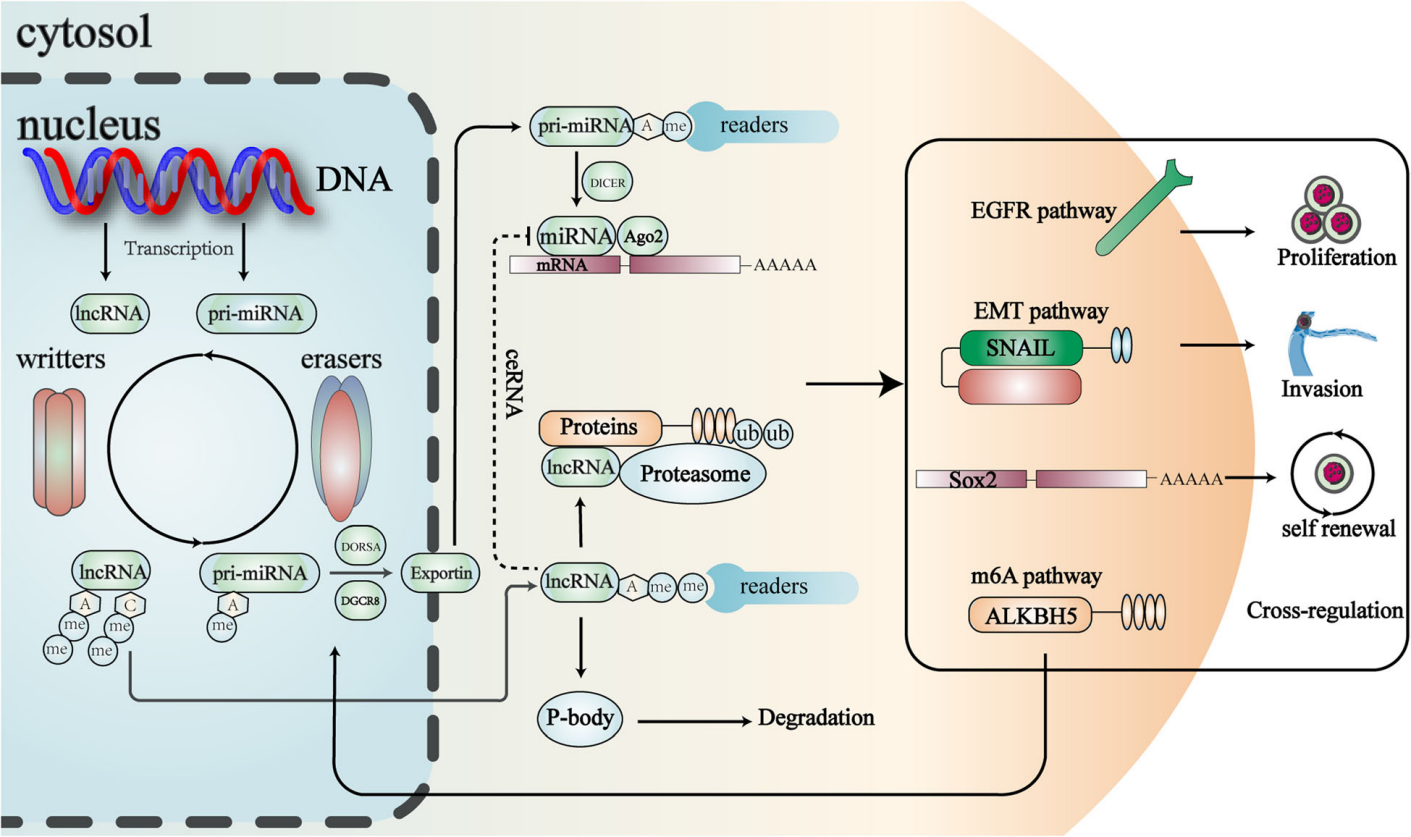
Epigenetic modulation of noncoding RNAs regulate cancer biology. Epigenetic modifications affect the metabolism and sublocation of ncRNA. m6A and m5C of lncRNA manipulate its stability and the binding with proteins and miRNAs (ceRNA). m6A of pri-miRNA regulates its processing procedure. As shown, the epigenetic modifications of ncRNA play crucial roles in regulating phenotypes of cancer cells by targeting members of various pathways. Moreover, lncRNA targets m6A eraser ALKBH5 to induce its ubiquitination and degradation, which means a cross-regulation between ncRNA and m6A pathway. A: adenosine, C: cytidine, me: methyl group

In summary, m6A related miRNAs and their downstream target genes are mainly involved in pathways of cell proliferation and metastasis, indicating that miRNA may be a critical bridge through which m6A regulates cancer progression (Fig. 1).

#### m6A regulates abundance and function lncRNAs

LncRNAs are now known to form a master controlling system at the transcriptional and post-transcriptional level, functioning as potent regulators in the process of tumorigenesis and cancer progression. Recently, transcriptome m6A-sequencing studies has mapped hundreds of m6A sites within lncRNAs, indicating a role of m6A in cancer biology through regulating lncRNAs.

Frequently, lncRNAs are upregulated in cancer tissues and act as oncogenes by promoting proliferative and metastatic ability of cancer cells. LncRNA RP11–138 J23.1 (*RP11*) promoted progression of colorectal cancer (CRC) by suppressing ubiquitination of ZEB1 protein and facilitating epithelial-mesenchymal transition (EMT) [26]. The high abundance of *RP11* in CRC cells were attribute to METTL3-mediated m6A methylation of *RP11*, which enhanced the stability of *RP11* transcript. In nasopharyngeal cancer, lncRNA *FAM225A* promoted NPC cell proliferation, migration and invasion by competitively absorbing miR-590-3p and miR-1275 to upregulate ITGB3 [68]. Moreover, bioinformatic analysis found two RRACU m6A sequence motifs in the last exon (position 2808 and 5460) of *FAM225A*. Subsequent functional assay showed METTL3-induced m6A modification of *FAM225A* improved its transcripts stability, which may partially account for the upregulation of *FAM225A* in NPC. On the contrary, some lncRNAs seemed to be more stable in low m6A level status. LncRNA *KCNK15-AS1* inhibited migration and invasion of pancreatic cancer cells by inhibiting EMT. The m6A eraser protein ALKBH5 can demethylated *KCNK15-AS1*, which resulted in the accumulation of *KCNK15-AS1* and impeded invasion of pancreatic cancer cells [69]. LncRNA nuclear paraspeckle assembly transcript 1 (*NEAT1*) promoted invasion and metastasis of gastric cancer cells by upregulating *EZH2*. ALKBH5 demethylated *NEAT1* and improved its stability, which eventually promoted metastasis of gastric cancer [70]. Previous study has identified at least 78 m6A sites in lncRNA X-inactive-specific transcript (*XIST*). The high abundance of m6A in *XIST* implied the importance of m6A in function of *XIST*. Consistently, METTL3 induced multi-m6A modification of *XIST*, which was indispensable for the its activity to silence target genes on X chromosome such as *Gpc4* and *Atrx* [29]. As *XIST* represented as a potent oncogene in colorectal cancer (CRC), our team presumed that m6A might regulate biological behavior of CRC cells through modifying *XIST*. As expected, we found that *XIST* was targeted by METTL14 instead of METTL3 in CRC cells. Moreover, RNA immunoprecipitation showed that m6A-*XIST* was recognized by reader protein YTHDF2 to induce the degradation of *XIST*, resulting in suppression of CRC progression (data unpublished).

The above studies suggested m6A decoration to lncRNAs could directly affect their stability and abundance, leading to reaction of downstream signaling. Moreover, the installed methyl group may affect the second structure of lncRNA, which significantly disturbs the interaction between lncRNAs and RNA binding proteins (RBPs) or miRNAs. The m6A modification of *lnc1281* enable it to sequester let-7 and promote differentiation of mouse embryonic stem cells (ESCs) [71]. Depletion or mutation of METTL3 abolished the m6A of *lnc1281* and interrupted the ceRNA network between *lnc1281* and let-7. Pseudogene *Olfr29-ps1* directly sponged miR-214-3p in myeloid-derived suppressor cells (MDSCs) to promote the immunosuppressive function and differentiation. Silencing METTL3 significantly abrogated the interaction of *Olfr29-ps1* and miR-214, indicating this ceRNA model is also m6A-dependent [72]. However, further investigation was needed to confirm the existence of similar mechanism in cancer cells. Collectively, m6A can either be a driving or suppressive force in the process of cancer progression. Although it is very typical for a regulatory system to have tissue and cellular specificity, there are other reasons for this issue. METTL3 and METTL14 may prefer different transcripts or different m6A sites in various cancers, which lead to distinct downstream signaling. The fate of m6A-modified lncRNAs also depends on the function of the different proteins that identify them, which may have impacts on stability, secondary structure, or subcellular localization. Moreover, in ceRNA regulatory model, the function of lncRNAs depends on the miRNA target. As m6A is a double-edged sword, the understanding of its involvement in tumorigenesis still needs further investigation.

Frequently being a “hunter”, sometimes m6A component can also be the “prey” of lncRNAs. Growth arrest special 5 antisense 1 (*GAS5-AS1*), the antisense lncRNA of the GAS5 gene, interacted with tumor suppressor gene GAS5, and increased its stability by recruiting ALKBH5 and demethylating GAS5 mRNA [73]. In glioblastoma cells, lncRNA *LOC100507424* (*FOXM1-AS*) acted in the similar way. *FOXM1-AS* promoted the interaction of ALKBH5 with FOXM1 nascent RNA, leading to demethylation and elevated expression of FOXM1 in glioblastoma stem-like cells, therefore enhancing self-renewal and tumorigenesis of glioblastoma [74]. In total, lncRNAs can use m6A as a powerful weapon to handle their targets, regulating target expression levels and malignant phenotypes of cancer cells (Fig. 1).

Recent studies showed that circRNAs played important roles in cancer progression mainly by functioning as “decoys” to neutralize miRNA or to sequester RNA binding proteins [75, 76]. Importantly, m6A motifs were also discovered in as many as hundreds of circRNAs [77, 78]. Functional assay showed that METTL3/14-induced m6A recruited translational initiation factor eIF4G2 to the start codon of the exons containing in circRNA, which promoted cap-independent translation of circRNAs [77, 79]. Intriguingly, m6A circRNAs are frequently derived from exons that are not methylated in mRNAs [78]. Moreover, about half of the m6A-circRNAs detected in HeLa cells were not detected in human embryonic stem cells, suggesting that many m6A-circRNAs are uniquely expressed in the cells [78]. However, as the function of m6A in circRNAs are largely unknown, the presence and function of m6A-circRNA in cancer cells need to be further validated.

### Other modifications of ncRNA and their effects on cancer progression

Other common RNA modifications include N1-methyladenosine (m1A), 5-methylcytidine (m5C), 7-methylguanosine (m7G) and pseudouridine (Ψ). Research concerning their functions and molecular mechanisms in human cancer has just begun. Besides, some modification patterns targeting tRNA and rRNA essentially regulate mRNA translation efficacy, which is not discussed in our review.

#### 2′-O-methylation (2′-O-me)

The primary transcript of canonical miRNAs is sequentially cleaved by the RNase III enzymes, Drosha and Dicer, which generate 5′ monophosphate end which is important for subsequent miRNA functions [17, 23]. In particular, the recognition of the 5′ monophosphate of pre-miRNAs by Dicer is crucial for precise and effective biogenesis of miRNAs. BCDIN3D is a human ortholog of the Bin3 family which is originally found in *Schizosaccharomyces pombe* and possesses putative S-adenosylMethionine (SAM) binding motif [80]. It O-methylates 5′ monophosphates of both DNA and RNA and prefers RNA to DNA because the deoxy-form of pre-miRNA is a far inferior substrate compared to its ribonucleotide counterpart. Methylation of the 5’monophosphate of pre-miR-145 by BCDIN3D neutralizes the negative charges of the 5′ monophosphate group. This abrogation of charge may prevent the association of pre-miR-145 with the Dicer, which result in the inhibition of the miRNA processing [81]. Consistently, knockdown of BCDIN3D leads to lower pre-miR-145 and concomitantly increased mature miR-145 levels in breast cancer cells, which suppresses their proliferative and invasive ability [81]. In total, this work also suggested O-methylation of miRNAs represents an important modulation of ncRNA and the 2′-O-methylation methyltransferase BCDIN3D might serve as a putative target in treatment of breast cancer.

Furthermore, several studies highlighted the potential clinical value of 2′-O-methylation by reporting that oral administration of 2′-O-methylated miRNAs resulted in the uptake of theses miRNAs into the intestine cells and subsequently regulated target mRNAs [82]. MiR-159 with a single 2′-O-methylation on the 3’end, which originated from plants, was found in human serum after oral administration [83]. Moreover, in vitro and in vivo assay revealed 2′-O-methylated miRNA-159 suppressed the proliferation of breast cancer cells, especially triple-negative BC cells, by targeting TCF-7 [84]. These results showed a promising blueprint of the cross-kingdom regulatory effects of plant miRNAs in mammals and suggested food-derived 2′-O-methylated miRNAs may serve as potential agents to improve current treatment for breast cancer.

#### 7-methylguanosine (m7G)

m7G is an essential modification at the 5′ cap of eukaryotic mRNA, regulating mRNA translation, sublocation and splicing [85, 86]. m7G also occurs internally within human cytoplasmic tRNA and 18S-rRNA, installed by METTL1-WDR4 [87], a m7G “writer” complex, which is similar to m6A complex, and WBSCR22 [88, 89], respectively. These internal m7G modifications impact RNA processing and function, especially mRNA translation [85, 90]. However, its existence and distribution within miRNAs or lncRNAs remain to be investigated. Using Borohydride Reduction sequencing (BoRed-seq) followed by mass spectrometry methods, Pandolfini, Barbieri, et al. showed that a subgroup of tumor suppressor miRNAs, including let-7e, contained m7G modification sites [91]. Subsequent assay showed that m7G methyltransferase METTL1 directly bind to the precursors of these miRNAs and accelerate their processing and maturation. Of course, this catalytic activity of METTL1 will influence the downstream target genes of miRNAs. Functional experiment showed that METTL1 inhibited cell migration and proliferation of lung cancer cells by downregulating HMGA2 in a let-7e dependent manner. Moreover, researchers found that only m7G of certain points (G11 of pri-let-7e) could lead to these alterations of RNA fate and cellular phenotype, indicating that m7G was not only a chemical but also a topological modification of RNA.

#### N1-methyladenosine (m1A), 5-methylcytidine (m5C) and pseudouridine (Ψ)

Similar to m6A, installing a methyl group at the N1 position of adenosine forms N1-methyladenosine (m1A). Nine members of m1A regulators have been identified till now including writers (TRMT6, TRMT61A, TRMT10C), readers (YTHDF1, YTHDC1) and erasers (ALKBH1, ALKBH3) [5, 92, 93]. Dysregulation of m1A components was found to promote the progression of gastric cancer and bladder cancer via activation of several oncogenic pathways such as PI3K/AKT/mTOR and ErbB Pathways [94]. However, whether m1A of ncRNAs were involved in these pathways remained obscure.

The modified base 5-methylcytosine (m5C) is well studied in DNA, investigations of its prevalence in cellular RNA have been largely confined to tRNA and rRNA [95, 96]. To date, many novel m5C sites have been validated by high-throughput techniques. A recent study using RNA bisulfite sequencing (RNA-BisSeq) performed with HeLa cells identified 5399 m5C sites within 2243 RNA molecules among which a majority (94%, 5063/5399) were found to occur within 1995 mRNAs [6]. The remaining 336 m5C sites were predominantly mapped to diverse types of lncRNAs, including pseudogene transcripts, lincRNAs and antisense transcripts. However, as bisulfite-seq may have a relatively high false positive rate, especially in double-strand RNA region, due to the resistance of double-strand transcripts to bisulfite treatment [97]. Analysis of a modified transcriptome-wide single base resolution sequencing called RBS-seq suggested 10-fold fewer total m5C sites in noncoding and coding RNAs even it detected hundreds of new m5C sites [98]. These results confirmed the existence of m5C in lncRNAs, even in a relatively low stoichiometry, suggesting the necessity of further validation.

Pseudouridine (Ψ), also known as the ‘fifth nucleotide’ in RNA, was first discovered in 1950s [99]. It has been known for decades that Ψ is present in tRNA, rRNA and snRNAs [100]. Recently, with the help of high-throughput sequencing method, Ψ was found to be present in mRNAs and lncRNAs as well. Several well-known lncRNAs, including *MALAT1* [100, 101], *PVT1* and *Kcnq1ot1* [101], were identified as targets of Ψ. Importantly, some of the Ψ sites were located within functional motifs of lncRNA, indicating the potential regulating effects of Ψ on lncRNAs [100]. However, the function and mechanism of Ψ in lncRNA and cancer progression remain to be elucidated.

#### Inosine (I)

Editing of adenosine to inosine (A-to-I), catalyzed by adenosine deaminases acting on RNA (ADARs), represents another pattern of posttranscriptional RNA modification. A-to-I editing can result in non-synonymous codon changes in transcripts as well as yield alternative splicing. Dysregulation of ADARs causes aberrant editing of its targets that may lead to cancer [102, 103]. Upregulation of ADAR1 was found in breast, lung, and esophageal cancer and promotes cancer progression [104, 105]. Moreover, it has been shown A-to-I editing also could affect the maturation and target specificity of miRNAs. For example, A-to-I editing close to the DROSHA cleavage site at + 3 on pri-let-7d impaired its biogenesis and reduced let-7d levels in chronic myelogenous leukemia, which resulted in malignant reprogramming of leukemia stem cells [106]. Edited miR-200b failed to inhibit EMT regulator ZEB1/ZEB2 and acquired new ability to repress a new group of targets such as leukemia inhibitory factor receptor (LIFR), promoting cell migration and invasion in a variety of cancer types [107]. In addition, edited form of lncRNA has also been shown to play a role in cancer progression. ADAR1-induced editing of lncRNA prostate cancer antigen 3 (PCA3) enhanced its ability of binding and suppressing PRUNE2 pre-mRNA, subsequently promoting cancer cell proliferation, adhesion and migration [108].

### Epigenetic modifications of several cancer-related ncRNAs

As above mentioned, recent studies have mapped the position of modified residues in single transcripts, identifying hundreds of ncRNAs, especially lncRNAs, with epigenetic modifications. In this section, we summarized several cancer-related miRNAs and lncRNAs carrying m6A and other epigenetic modulations and the assessed potential roles of these modified-ncRNAs in human cancer.

#### m6A regulated mature miRNA

In addition to the DGCR8-mediated m6A methylation to pri-miRNAs, a recent study discovered that a group of mature miRNAs, including miR-17-5p, − 21-5p, and -200c-3p and let-7a-5p, also harbor methyl residues [109]. Let-7a-5p and miR-17-5p had m6A whereas miR-200c-3p and miR-21-3p had m5C modifications at specific positions in the mature sequence which potentially altered their stability and target recognition ability. In miR-200c-3p, the m5C modification at position 9 cytosine (C9) was close to RNA recognition bases. Methyl groups installed at C9 disrupted hydrogen bonding between miRNA and Ser220 of Argonaute (AGO) protein, leading to a positional shift of the guanine at position 8 [109]. In miR-17-5p and let-7a-5p, m6A caused drastic structural change of the whole miRNA sequence, including that around the RNA recognition site, which affected the target RNA recognition efficiency. These findings indicated that m6A and m5C of mature miRNAs may reduce its the ability to suppress the abundance or translation of target mRNA [109]. More importantly, the methylation levels of these miRNAs in pancreatic and colorectal cancer tissues were significantly higher than paired normal samples whereas no differences in miRNA expression level were detected. Furthermore, miR-17-5p methylation level in serum samples distinguished early pancreatic cancer patients from healthy controls with higher sensitivity and specificity than established biomarkers such as carbohydrate antigen 19–9 (CA19–9) and carcinoembryonic antigen (CEA) [109]. These results suggested the clinical relevance of miRNA epigenetic modulations and provided a novel diagnostic strategy for early-stage cancer. However, the m6A and m5C methyltransferases which mediated these modifications were not identified. The alteration of the downstream target mRNAs and the effects on malignant phenotypes of cancer cells of these miRNAs modulations was still needed to be explored in detail.

#### Metastasis associated lung adenocarcinoma transcript 1 (MALAT1)

Metastasis associated lung adenocarcinoma transcript 1 (*MALAT1*), also known as nuclear-enriched abundant transcript 2 (*NEAT2*), is a highly conserved and lncRNA of ~ 8 kb in size which is ubiquitously expressed in cancer and normal tissues [110, 111]. *MALAT1* has been found to manipulate proliferation, migration and apoptosis in many different human cancers such as pancreatic cancer, lung cancer and ovarian cancer [112, 113]. A recent study reported *MALAT1* was a tumor suppressor which impaired metastatic ability of breast cancer cells by binding and inactivating oncogene TEAD, calling for rectification of the effects of this classical prometastatic lncRNA [114]. Researchers attributed the contradictory results to the loss of adjacent regulating genes (including *Neat1*, *Frmd8*, *Tigd3*, et al.) resulted from previous *Malat1* deletion model. However, there may be other possible answers to this question. Previous MeRIP-Seq assay identified at least seven m6A peaks in *MALAT1*. Importantly, secondary structure prediction and mapping experiments demonstrated that two residues (A2515 and A2577) are located in hairpin stems of *MALAT1*, which suggest m6A of these two sites might lead to change of structure [4, 8]. Subsequent mapping experiment showed m6A of A2577 destabilizes the hairpin stem of *MALAT1*, making it accessible for RNA-binding proteins such as HNRNPC for recognition [57]. These results support the hypothesis that m6A regulates protein binding through its influence on structure of lncRNA. It is reasonable to presume that structure changes induced by m6A modifications may apply to a larger family of m6A-regulated RNA. In addition, *MALAT1* also possesses several putative m5C and pseudouridine residues [100, 115]. However, enzymes responsible for these modifications and their impact on molecular function of *MALAT1* are still unknown.

#### The Hox transcript antisense intergenic RNA (HOTAIR)

The Hox transcript antisense intergenic RNA (*HOTAIR*) is an intergenic lncRNA of which is transcribed from the antisense strand of the developmental HOXC gene cluster on chromosome 12 [116]. Abnormal expression of *HOTAIR* exists in various human cancers including melanoma, hepatocellular carcinoma, gastric cancer, colorectal cancer and pancreatic cancer [117–120]. Moreover, the expression level of *HOTAIR* is proved to be correlated with cancer progression and unfavorable clinical outcomes of cancer patients in most solid cancers. Given the fact that *HOTAIR* is a pivotal regulator and promising target, modifications of *HOTAIR* may have profound influence on cancer progression. Mechanically, *HOTAIR* can bind to histone H3 lysine 27 (H3K27) methylase complex, PRC2 and histone demethylase complex, lysine specific demethylase 1 (LSD1) to induce to induce epigenetic gene silencing [121, 122]. Intriguingly, a recent study identified a specific cytosine methylation in *HOTAIR* at position 1683 (C1683), located within the LSD1 binding motif [123]. Therefore, it is logical to speculate a regulatory impact of epitranscriptome on epigenome. Moreover, eight m6A sites were identified along the *HOTAIR* sequence, indicating a potential effect of m6A on the function of m6A [29]. However, till now, methylation-dependent interaction between *HOTAIR* and LSD1 with downstream effects on cancer progression has not been established.

Other lncRNAs identified with epigenetic modulation residues including SRA1, GAS5, TERC, TUG1, ANRIL, and several SNHG family members were summarized in Table 1. Nevertheless, the function of these modified lncRNAs in cancers are largely undermined and require further validation.

**Table 1:** ncRNAs with epigenetic modulations

Name	Category	m6A	m5C	Ψ
*let-7a-5p*	miRNA	1	NA	NA
*miR-17-5p*	miRNA	1	NA	NA
*miR-200c-3p*	miRNA	NA	1	NA
*miR-21-5p*	miRNA	NA	1	NA
*XIST*	lncRNA	78	5	1
*MALAT1*	lncRNA	3	7	3
*PVT1*	lncRNA	2	1	1
*NEAT1*	lncRNA	2	7	NA
*HOTAIR*	lncRNA	1	1	NA
*SRA1*	lncRNA	4	NA	1
*ANRIL*	lncRNA	1	2	NA
*GAS5*	lncRNA	NA	2	NA
*SNHG1*	lncRNA	NA	NA	1
*SNHG7*	lncRNA	NA	NA	1
*SNHG12*	lncRNA	NA	NA	2
*TUG1*	lncRNA	11	NA	NA
*TERC*	lncRNA	NA	3	6
*RPPH1*	lncRNA	NA	4	NA

*NA* not available. Only lncRNAs with multiple modulations were shown

### Clinical relevance of RNA epigenetic modulation

Given the diagnostic and prognostic value of ncRNAs, it is tempting to assume that agonists or inhibitors of epigenetic modulation enzymes may provide potential therapeutic targets for cancers. As a matter of fact, many recombinant compounds targeting m6A pathway have already been under pre-clinical evaluation. We summarized the existing drugs tested in human cancer models to get a prospect of further clinical application. Meclofenamic acid 2 (MA2), a selective inhibitor of FTO, exerted substantial inhibitory effects on the growth of glioblastoma stem-like cells (GSC) [124] and acute myeloid leukemia (AML) [125] in mice xenograft model. R-2-hydroxyglutarate (R-2HG), produced by mutant isocitrate dehydrogenase 1/2 (IDH1/2) enzymes, was once regarded as an oncometabolite. Recent study found that R-2HG inhibited FTO activity, thereby increasing global m6A level, which in turn decreased the stability of *MYC* and suppressed leukemia progression both in vitro and in vivo [126]. METTL3 methylated and stabilized *SOX2* mRNA, promoting self-renewal and metastasis of CRC cells. Patient derived xenograft (PDX) model was established to evaluate the potential therapeutic effect of METTL3. Surprisingly, intratumor injection of siMETTL3 significantly impeded the tumor growth compared to control group treated with placebo, indicating a promising therapeutic strategy based on efficient inhibitors of METTL3 for CRC [25]. Besides, many other compounds targeting m6A demethylases were identified, but their effects were rarely explored in cancer cell models (Table 2) [127–131]. Therefore, novel therapeutic strategies based on m6A methylation should be further validated in animal models and clinical trials.

**Table 2:** Current developed inhibitors of RNA epigenetic modulation enzymes

Compounds	Targets	Cancer model	Reference
MA/MA2	FTO	Glioblastoma xenografted mice	Cui Q, Shi H, et al. [124]
R-2HG	FTO	Leukemia xenograft mice model	Su R, Dong L, et al. [126]
FB23/FB23–2	FTO	Leukemia xenograft mice model	Huang Y, Su R, et al. [125]
siMETTL3	METTL3	Colorectal cancer PDX model	Li T, Hu PS, et al. [25]
N-CDPCB	FTO	3 T3-L1 cells	He W, Zhou B, et al. [127]
CHTB	FTO	3 T3-L1 cells	Qiao Y, Zhou B, et al. [128]
Citrate	ALKBH5	None	Xu C, Liu K, et al. [129]
FG-2216	FTO ALKBH5	None	Aik W, Demetriades M, et al. [130] Aik W, Scotti JS,et al. [131]

*PDX* patient-derived xenograft

## Conclusions and future perspectives

Our study summarized the current advances of epigenetic modulation of ncRNAs (termed as “ncRNA epigenetics” or “ncRNA epitranscriptomics”) and provided an attractive regulatory mechanism of cancer progression, unveiling a novel dimension of cancer biology. In summary, ncRNA epigenetics is involved in almost every step of RNA metabolism, regulating stability of ncRNAs, miRNA processing, ceRNA network, as well as affecting interaction between lncRNAs and RBPs. Moreover, several key enzymes in the cascade of epitranscriptomic reactions seem to be determinant for cancer progression and may represent potential therapeutic targets. Although some m6A inhibitors have showed promising effects in several types of cancer, more selective and powerful drugs are expected to be explored. Besides, the side effects of those inhibitors should also be evaluated, for RNA epigenetic modulation influences gene expression in many aspects. In brief, we are only starting to unravel the full breadth of this research field, the underlying mechanisms of ncRNA epigenetics in human cancer should be further addressed.

## Data Availability

All data generated or analyzed during this study are included in this published article and its additional files.

## Abbreviations

2′-O-Me: 2′-O-methylation
A to I: Adenosine to inosine
ADAR: Adenosine deaminases acting on RNA
ALKBH5: Alkylation repair homolog protein 5
AML: Acute myeloid leukemia
circRNA: Circular RNA
EIF3: Eukaryotic initiation factor 3
ESC: Embryonic stem cell
FTO: Fat mass- and obesity-associated protein
GAS5-AS1: Growth arrest special 5 antisense 1
GC: Gastric cancer
GSC: Glioblastoma stem-like cells
HCC: Hepatocellular carcinoma
HNRNP: Heterogeneous nuclear ribonucleoprotein
HOTAIR: The Hox transcript antisense intergenic RNA
IDH1/2: Isocitrate dehydrogenase 1/2
IGF2BP: Insulin like growth factor 2 mRNA binding protein
LIFR: Leukemia inhibitory factor receptor
lncRNA: Long noncoding RNA
LSD1: Lysine specific demethylase 1
m1A: N1-methyladenosine
m5C: 5-methylcytidine
m6A: N6-methyladenosine
m7G: 7-methylguanosine
MA2: Meclofenamic acid 2
MALAT1: Metastasis associated lung adenocarcinoma transcript 1
MCT: M6a methyltransferase complex
MDSC: Myeloid-derived suppressor cell
METTL14: Methyltransferase-like 14
METTL3: Methyltransferase-like 3
miRNA: Microrna
NEAT1: Nuclear paraspeckle assembly transcript 1
NEAT2: Nuclear-enriched abundant transcript 2
PCA3: Prostate cancer antigen 3
PDX: Patient derived xenograft
R-2HG: R-2-hydroxyglutarate
RBM: RNA binding motif
RBM15: RNA-binding motif protein 15
RBP: RNA binding protein
rRNA: Ribosomal RNA
SAM: S-adenosylmethionine
tRNA: Transfer RNA
UTR: Untranslated terminal region
WTAP: Wilms tumor 1-associated protein
XIST: X-inactive-specific transcript
YTHDF: YT521-B homology domain-containing protein family
